# 4-Amino-3,5-dichloro­benzene­sulfonamide

**DOI:** 10.1107/S1600536810040584

**Published:** 2010-10-20

**Authors:** Xiu-Ying Qin, Han-Fu Liu, Jia-Xun Lin

**Affiliations:** a College of Pharmacy, Guilin Medical University, Guilin, Guangxi 541004, People’s Republic of China

## Abstract

In the title compound, C_6_H_6_Cl_2_N_2_O_2_S, the O atoms of the sulfonamide group lie on one side of the benzene ring and the amino group lies on the opposite side. An inter­molecular N—H⋯Cl inter­action occurs. In the crystal, adjacent mol­ecules are linked by N—H⋯O hydrogen bonds, forming a three-dimensional structure with supporting π–π stacking inter­actions [centroid–centroid distance = 3.7903 (12) Å]. A short Cl⋯Cl contact [3.3177 (10) Å] also occurs.

## Related literature

For the preparation, see: Qiu & Lv (2005 ▶). For Cl⋯Cl contacts, see: Sakurai *et al.* (1963 ▶); Stone *et al.* (1994 ▶); Qin *et al.* (2008 ▶).
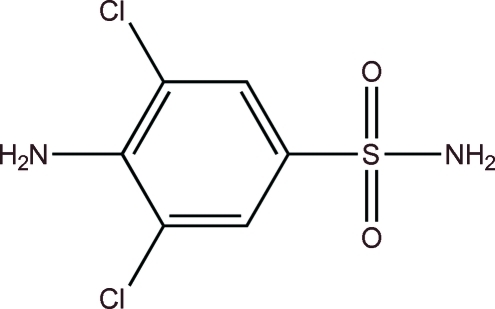


## Experimental

### 

#### Crystal data

C_6_H_6_Cl_2_N_2_O_2_S
*M* *_r_* = 241.09
Monoclinic, 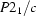

*a* = 8.9544 (17) Å
*b* = 13.387 (3) Å
*c* = 7.5673 (15) Å
β = 95.809 (2)°
*V* = 902.4 (3) Å^3^
*Z* = 4
Mo *K*α radiation
μ = 0.92 mm^−1^
*T* = 93 K
0.30 × 0.27 × 0.13 mm

#### Data collection

Rigaku SPIDER diffractometer
Absorption correction: multi-scan (*ABSCOR*; Higashi, 1995 ▶) *T*_min_ = 0.771, *T*_max_ = 0.888
5918 measured reflections
2008 independent reflections
1759 reflections with *I* > 2σ(*I*)
*R*_int_ = 0.020

#### Refinement

*R*[*F* ^2^ > 2σ(*F* ^2^)] = 0.029
*wR*(*F* ^2^) = 0.074
*S* = 1.00
2008 reflections
134 parameters
H atoms treated by a mixture of independent and constrained refinement
Δρ_max_ = 0.45 e Å^−3^
Δρ_min_ = −0.36 e Å^−3^

### 

Data collection: *RAPID-AUTO* (Rigaku, 2004 ▶); cell refinement: *RAPID-AUTO*; data reduction: *RAPID-AUTO*; program(s) used to solve structure: *SHELXS97* (Sheldrick, 2008 ▶); program(s) used to refine structure: *SHELXL97* (Sheldrick, 2008 ▶); molecular graphics: *SHELXTL* (Sheldrick, 2008 ▶); software used to prepare material for publication: *SHELXL97*.

## Supplementary Material

Crystal structure: contains datablocks I, global. DOI: 10.1107/S1600536810040584/nk2060sup1.cif

Structure factors: contains datablocks I. DOI: 10.1107/S1600536810040584/nk2060Isup2.hkl

Additional supplementary materials:  crystallographic information; 3D view; checkCIF report

## Figures and Tables

**Table 1:** Hydrogen-bond geometry (Å, °)

*D*—H⋯*A*	*D*—H	H⋯*A*	*D*⋯*A*	*D*—H⋯*A*
N1—H1*A*⋯Cl1	0.80 (2)	2.60 (2)	2.9793 (18)	111 (2)
N1—H1*B*⋯O2^ii^	0.83 (3)	2.40 (3)	3.199 (2)	160 (2)
N2—H2*A*⋯O1^i^	0.81 (2)	2.14 (2)	2.935 (2)	167 (2)
N2—H2*B*⋯O2^iii^	0.86 (3)	2.08 (3)	2.934 (2)	172 (2)

Symmetry codes: (i) 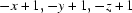; (ii) 
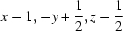
; (iii) 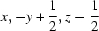.

**Fig. 1.**
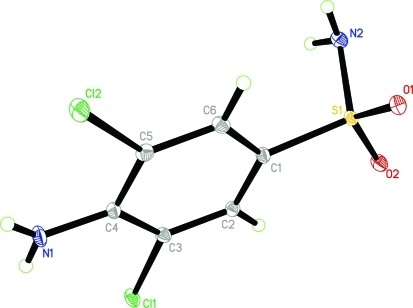
The molecular structure of (I), with atom labels and 30% probability displacement ellipsoids.

**Fig. 2.**
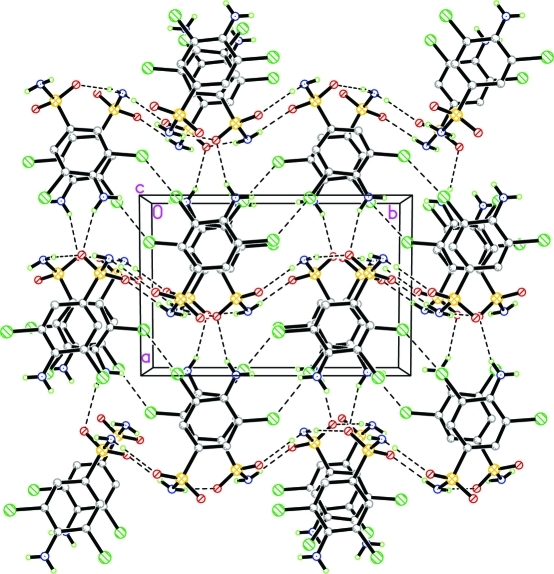
The packing of (I), viewed down the c axis, showing layers of molecules conneted by N—H···O hydrogen bonds, Cl···Cl contact and π–π stacking interactions.

## supplementary crystallographic information

### Comment

4-Amino-3,5-dichloro-benzenesulfonamide is a primary substance used to synthesize Diclofenac sodium. Although the synthetic methods of the title compound has been reported (Qiu & Lv, 2005), no crystal structure data have been published so far. Our experimental aim was to obtain a Co(II) complex by preparing the potassium salt of 4-amino-3,5-dichloro-benzenesulfonamide modified by salicylaldehyde, and then reacting with CoCl_2_.6H_2_O  To our surprise, we obtained yellow crystals which were identified as the title compound. Here, we report the synthesis and crystal structure of the title compound.

In the title compound, C_6_H_6_Cl_2_N_2_O_2_S, the amino and chlorine substituent groups are essentially co-planar (maximum r.m.s. deviation of fitted atoms is -0.0747 Å) with the benzene ring to which they are bonded. The O atoms of the sulfonamide group lie on one side of the benzene ring and the amino group lies on the opposite side (Scheme I, Fig. 1)

In the crystal, molecules are linked by intermolecular hydrogen bonds N—H···O (Fig.2., Table 2) and π–π stacking interactions [3.790 Å, the distance of the centroids between the two benzene rings, symmetry code as *x*, -*y* + 1/2, *z* + 1/2 and *x*, -*y* + 1/2, *z* - 1/2], and a short Cl···Cl contact [3.318 Å, symmetry code as -*x*, -*y* - 1, -*z*] occurs (Fig. 2). The Cl···Cl contact is stronger than that of the [Zn(II)(C_8_H_7_O_4_NCl_2_S)(Phen)(H_2_O)]_3 _(Qin *et al.*, 2008).

### Experimental

Powdered 4-amino-3,5-dichloro-benzenesulfonamide (2.671 g, 10.08 mmol), KOH(0.504 g, 9.0 mmol) and salicylaldehyde (0.53 ml, 5.01 mmol) were mixed in methanol (40 ml). The mixture was stirred at 323 K for 2 h. Then, 1.30 ml solution was taken out from the mixed solution was added into a solution of CoCl_2_.6H_2_O(0.060 g, 0.25 mmol) in methanol (25 ml). This mixture has been stirred at 323 K for 8 h. Subsequently, the reagents were filtrated. The resulting solution was left at room temperature, orange-yellow crystals were obtained after some days.

### Refinement

All H atoms were geometrically positioned and refined using a mixed model, with distance restraints of C—H = 0.9500 and N—H = 0.80 (2)–0.86 (3) Å, and with*U*_iso_(H) = 1.2*U*_eq_(C).

### Crystal data

**Table:**

C_6_H_6_Cl_2_N_2_O_2_S	*F*(000) = 488
*M**_r_* = 241.09	*D*_x_ = 1.774 Mg m^−^^3^
Monoclinic, *P*2_1_/*c*	Melting point = 207.2–207.5 K
Hall symbol: -P 2ybc	Mo *K*α radiation, λ = 0.71073 Å
*a* = 8.9544 (17) Å	Cell parameters from 2008 reflections
*b* = 13.387 (3) Å	θ = 3.0–27.5°
*c* = 7.5673 (15) Å	µ = 0.92 mm^−^^1^
β = 95.809 (2)°	*T* = 93 K
*V* = 902.4 (3) Å^3^	Block, orange-yellow
*Z* = 4	0.30 × 0.27 × 0.13 mm

### Data collection

**Table:**

Rigaku SPIDER diffractometer	2008 independent reflections
Radiation source: rotating anode	1759 reflections with *I* > 2σ(*I*)
graphite	*R*_int_ = 0.020
ω scans	θ_max_ = 27.5°, θ_min_ = 3.0°
Absorption correction: multi-scan (*ABSCOR*; Higashi, 1995)	*h* = −10→10
*T*_min_ = 0.771, *T*_max_ = 0.888	*k* = −17→10
5918 measured reflections	*l* = −9→9

### Refinement

**Table:**

Refinement on *F*^2^	Primary atom site location: structure-invariant direct methods
Least-squares matrix: full	Secondary atom site location: difference Fourier map
*R*[*F*^2^ > 2σ(*F*^2^)] = 0.029	Hydrogen site location: inferred from neighbouring sites
*wR*(*F*^2^) = 0.074	H atoms treated by a mixture of independent and constrained refinement
*S* = 1.00	*w* = 1/[σ^2^(*F*_o_^2^) + (0.0461*P*)^2^ + 0.260*P*] where *P* = (*F*_o_^2^ + 2*F*_c_^2^)/3
2008 reflections	(Δ/σ)_max_ = 0.001
134 parameters	Δρ_max_ = 0.45 e Å^−^^3^
0 restraints	Δρ_min_ = −0.36 e Å^−^^3^

### Special details

**Table:**

Geometry. All e.s.d.'s (except the e.s.d. in the dihedral angle between two l.s. planes) are estimated using the full covariance matrix. The cell e.s.d.'s are taken into account individually in the estimation of e.s.d.'s in distances, angles and torsion angles; correlations between e.s.d.'s in cell parameters are only used when they are defined by crystal symmetry. An approximate (isotropic) treatment of cell e.s.d.'s is used for estimating e.s.d.'s involving l.s. planes.
Refinement. Refinement of *F*^2^ against ALL reflections. The weighted *R*-factor *wR* and goodness of fit *S* are based on *F*^2^, conventional *R*-factors *R* are based on *F*, with *F* set to zero for negative *F*^2^. The threshold expression of *F*^2^ > σ(*F*^2^) is used only for calculating *R*-factors(gt) *etc*. and is not relevant to the choice of reflections for refinement. *R*-factors based on *F*^2^ are statistically about twice as large as those based on *F*, and *R*– factors based on ALL data will be even larger.

### Fractional atomic coordinates and isotropic or equivalent isotropic displacement parameters (Å^2^)

**Table:**

	*x*	*y*	*z*	*U*_iso_*/*U*_eq_	
Cl1	0.24595 (5)	0.01031 (3)	0.45203 (6)	0.01881 (13)	
Cl2	0.00000 (5)	0.37179 (3)	0.29728 (7)	0.02268 (13)	
S1	0.57265 (5)	0.34844 (3)	0.58837 (6)	0.01325 (12)	
O1	0.53529 (14)	0.43437 (9)	0.68889 (17)	0.0171 (3)	
O2	0.67408 (13)	0.27471 (9)	0.67149 (18)	0.0190 (3)	
N1	0.00496 (18)	0.15006 (13)	0.3074 (2)	0.0198 (4)	
N2	0.64946 (18)	0.39096 (12)	0.4206 (2)	0.0166 (3)	
C1	0.40449 (19)	0.28746 (13)	0.5127 (2)	0.0135 (3)	
C2	0.39422 (19)	0.18356 (12)	0.5117 (2)	0.0137 (3)	
H2	0.4778	0.1437	0.5551	0.016*	
C3	0.26064 (19)	0.13958 (13)	0.4466 (2)	0.0145 (4)	
C4	0.13407 (19)	0.19420 (13)	0.3762 (2)	0.0143 (4)	
C5	0.15166 (19)	0.29916 (13)	0.3806 (2)	0.0155 (4)	
C6	0.28195 (19)	0.34575 (13)	0.4475 (2)	0.0151 (4)	
H6	0.2885	0.4166	0.4493	0.018*	
H1A	−0.001 (3)	0.0906 (19)	0.305 (3)	0.032 (7)*	
H2A	0.604 (3)	0.4378 (17)	0.375 (3)	0.022 (6)*	
H1B	−0.071 (3)	0.1841 (18)	0.277 (3)	0.033 (7)*	
H2B	0.665 (3)	0.3444 (19)	0.346 (4)	0.039 (7)*	

### Atomic displacement parameters (Å^2^)

**Table:**

	*U*^11^	*U*^22^	*U*^33^	*U*^12^	*U*^13^	*U*^23^
Cl1	0.0172 (2)	0.0123 (2)	0.0260 (2)	−0.00223 (16)	−0.00262 (17)	−0.00010 (16)
Cl2	0.0118 (2)	0.0194 (2)	0.0355 (3)	0.00248 (17)	−0.00420 (18)	0.00595 (19)
S1	0.0103 (2)	0.0123 (2)	0.0165 (2)	−0.00140 (15)	−0.00131 (16)	0.00062 (15)
O1	0.0175 (7)	0.0159 (6)	0.0177 (6)	−0.0018 (5)	0.0006 (5)	−0.0024 (5)
O2	0.0132 (7)	0.0156 (6)	0.0267 (7)	−0.0008 (5)	−0.0049 (5)	0.0048 (5)
N1	0.0123 (9)	0.0168 (8)	0.0287 (9)	−0.0024 (7)	−0.0059 (7)	0.0018 (7)
N2	0.0154 (8)	0.0138 (7)	0.0207 (8)	−0.0003 (6)	0.0027 (6)	−0.0002 (6)
C1	0.0112 (9)	0.0153 (8)	0.0137 (8)	−0.0015 (6)	0.0005 (7)	−0.0005 (7)
C2	0.0120 (9)	0.0142 (8)	0.0146 (8)	0.0005 (7)	0.0003 (7)	0.0006 (7)
C3	0.0152 (9)	0.0123 (8)	0.0160 (8)	−0.0006 (7)	0.0017 (7)	0.0008 (6)
C4	0.0113 (9)	0.0174 (8)	0.0140 (8)	−0.0019 (7)	0.0003 (7)	0.0004 (7)
C5	0.0123 (9)	0.0168 (8)	0.0172 (9)	0.0036 (7)	0.0007 (7)	0.0036 (7)
C6	0.0138 (9)	0.0120 (8)	0.0195 (9)	0.0004 (6)	0.0020 (7)	0.0008 (7)

### Geometric parameters (Å, °)

**Table:**

Cl1—C3	1.7363 (18)	N2—H2B	0.86 (3)
Cl2—C5	1.7360 (17)	C1—C2	1.394 (2)
S1—O1	1.4370 (13)	C1—C6	1.395 (2)
S1—O2	1.4419 (13)	C2—C3	1.379 (2)
S1—N2	1.6078 (16)	C2—H2	0.9500
S1—C1	1.7571 (18)	C3—C4	1.407 (2)
N1—C4	1.355 (2)	C4—C5	1.414 (2)
N1—H1A	0.80 (2)	C5—C6	1.374 (2)
N1—H1B	0.83 (3)	C6—H6	0.9500
N2—H2A	0.81 (2)		
			
O1—S1—O2	119.15 (8)	C3—C2—C1	118.85 (16)
O1—S1—N2	106.00 (8)	C3—C2—H2	120.6
O2—S1—N2	106.61 (8)	C1—C2—H2	120.6
O1—S1—C1	107.86 (8)	C2—C3—C4	123.34 (16)
O2—S1—C1	107.78 (8)	C2—C3—Cl1	118.83 (13)
N2—S1—C1	109.15 (8)	C4—C3—Cl1	117.83 (13)
C4—N1—H1A	119.6 (18)	N1—C4—C3	122.82 (17)
C4—N1—H1B	120.7 (17)	N1—C4—C5	122.09 (16)
H1A—N1—H1B	119 (2)	C3—C4—C5	115.08 (15)
S1—N2—H2A	112.2 (16)	C6—C5—C4	123.25 (16)
S1—N2—H2B	111.9 (17)	C6—C5—Cl2	118.92 (14)
H2A—N2—H2B	113 (2)	C4—C5—Cl2	117.83 (13)
C2—C1—C6	120.47 (16)	C5—C6—C1	118.99 (16)
C2—C1—S1	121.28 (13)	C5—C6—H6	120.5
C6—C1—S1	118.21 (13)	C1—C6—H6	120.5
			
O1—S1—C1—C2	−140.05 (14)	Cl1—C3—C4—N1	−2.6 (2)
O2—S1—C1—C2	−10.20 (17)	C2—C3—C4—C5	−1.5 (3)
N2—S1—C1—C2	105.21 (15)	Cl1—C3—C4—C5	178.10 (13)
O1—S1—C1—C6	42.09 (16)	N1—C4—C5—C6	−179.13 (17)
O2—S1—C1—C6	171.94 (13)	C3—C4—C5—C6	0.2 (3)
N2—S1—C1—C6	−72.65 (16)	N1—C4—C5—Cl2	1.0 (2)
C6—C1—C2—C3	−0.5 (3)	C3—C4—C5—Cl2	−179.65 (13)
S1—C1—C2—C3	−178.35 (13)	C4—C5—C6—C1	0.8 (3)
C1—C2—C3—C4	1.7 (3)	Cl2—C5—C6—C1	−179.29 (13)
C1—C2—C3—Cl1	−177.91 (13)	C2—C1—C6—C5	−0.7 (3)
C2—C3—C4—N1	177.82 (17)	S1—C1—C6—C5	177.19 (13)

### Hydrogen-bond geometry (Å, °)

**Table:**

*D*—H···*A*	*D*—H	H···*A*	*D*···*A*	*D*—H···*A*
N1—H1A···Cl1	0.80 (2)	2.60 (2)	2.9793 (18)	111 (2)
N2—H2A···O1^i^	0.81 (2)	2.14 (2)	2.935 (2)	167 (2)
N1—H1B···O2^ii^	0.83 (3)	2.40 (3)	3.199 (2)	160 (2)
N2—H2B···O2^iii^	0.86 (3)	2.08 (3)	2.934 (2)	172 (2)

Symmetry codes: (i) −*x*+1, −*y*+1, −*z*+1; (ii) *x*−1, −*y*+1/2, *z*−1/2; (iii) *x*, −*y*+1/2, *z*−1/2.
